# Recommendations of using at least two different methods for measuring NO

**DOI:** 10.3389/fpls.2013.00058

**Published:** 2013-03-20

**Authors:** Kapuganti J. Gupta, Abir U. Igamberdiev

**Affiliations:** ^1^Department of Plant Sciences, University of OxfordOxford, UK; ^2^Department of Biology, Memorial University of Newfoundland, St. John'sNL, Canada

## Introduction

Nitric oxide (NO) is widely recognized as a signal molecule in plants. Various sources of NO were identified in plants (Moreau et al., 2010; Gupta et al., 2011; Mur et al., 2013) located in different compartments activated under various conditions. Briefly these are the mitochondrial nitrite: NO reductase reaction, the cytosolic nitrate reductase (NR), the plasma membrane nitrite: NO reductase (PM-NiNOR), xanthine oxidoreductase, NO synthase-like enzyme (putative), polyamine (PA)- and hydroxylamine (HA)-mediated pathways. NO acts as an intracellular messenger due to its diffusible capacity through various cellular compartments. After pioneering discovery of NO production in plants, scientists started digging deeply to the key function of NO and this research led to understanding of various roles of NO that include regulation of stomatal movement, root development, floral transition, response to biotic and abiotic stresses, symbiotic interactions. Despite of extensive research on NO roles in metabolism and signal transduction, its measurement remains challenging (Vandelle and Delledonne, 2008; Mur et al., 2011). We discuss below the problems associated with NO measurement and suggest some important solutions to tackle these problems.

The half-life of NO depends on its concentration and usually falls is in the range of ten(s) seconds. For instance, at 10 μM concentration NO has a half-life of about 80 s whereas at 100 μM concentration NO has a half-life of about 8 s (Wink and Mitchell, 1998). This means that at low concentration NO can easily diffuse from its origin to the site of action. At higher concentrations most of the NO rapidly undergoes autoxidation. Moreover NO half-life also depends on the presence of scavengers such as non-symbiotic hemoglobin (class 1) and other major NO scavenging targets such as lipids and metal-containing proteins. Therefore, it is often very important to measure NO concentration which is crucial for understanding its function. But NO concentrations measured in same biological material by different methods often give different values (Mur et al., 2006; Planchet and Kaiser, 2006). In some cases NO production is localized to specific cells such as guard cells; therefore fluorescent probes are required to visualize NO producing sites. Here we describe why we need to use at least two different methods for measuring generation of NO in plants and suggest the best combination of methods for specific studies.

## Overview of NO detection methods: advantages and technical problems

### Measurement of NO in the gas phase

Chemiluminescence is a well-established method for NO measurement. In this method the reaction between NO and ozone (O_3_) generates nitrogen dioxide (NO^*^_2_) in the excited-state which then emits a photon and reaches its ground state, a photomultiplier counts the light generated in the amount proportional to NO content. Chemiluminescence has been used to measure NO emissions from leaves, roots and from isolated mitochondria (Planchet et al., 2005; Gupta et al., 2011; Shah et al., 2013). This method is highly sensitive and can detect NO in the range of parts per billion which corresponds to picomolar concentrations in the tissue. The major disadvantage of this method is that it measures only the emitted NO in the gas phase (Planchet and Kaiser, 2006). Another disadvantage is that it measures only pure NO emitted from biological samples, while only a small portion (in green leaves of Arabidopsis less than 6%) of the produced NO is emitted from the biological samples, the major part is quenched in the reaction with superoxide (Vanin et al., 2004), and in the hypoxic tissues scavenged by the non-symbiotic hemoglobin (Igamberdiev et al., 2006).

Laser-based photoacoustic detection of NO uses the absorption of rapidly chopped infrared light by NO (Mur et al., 2011). The sound is generated during the absorption and relaxation which is detected by the microphone located in the photoacoustic cell. This method was used by Mur et al. (2005) to detect NO from tobacco leaves infected with bacteria (Pseudomonas). The advantage of this method is it very high precision, while the disadvantage is that NO can be detectable only in the gas phase and no special information about NO production in specific cells can be obtained. This method should be used together with DAF fluorescence (see below) to get the information about the presence of NO in the gas phase and in specific cells respectively.

Quantum cascade laser-based spectroscopic detection of NO (Moeskops et al., 2006; Gupta et al., 2013) represents another version of the previous method. The laser is integrated with a thermo-electrically cooled infrared detector. The detection sensitivity of this method is 0.03 parts per billion which is higher by 1–2 orders of magnitude than in the photoacoustic detection.

The Membrane Inlet Mass Spectrometry (MIMS) is a robust method for NO detection (Conrath et al., 2004). In this method the online detection of NO in gaseous phase is possible. The membrane barrier separates the sample from mass spectrometer and allows NO to be detected. Another advantage of MIMS is that, by using radioactive substrates, it is possible to detect the contribution of NO from each pathway by using radiolabelled arginine or nitrate/nitrite (Conrath et al., 2004). Though it is an excellent and very sensitive method, it was rarely used in plant NO research. This is probably due to its high cost and expertise requirement. In this method, as in other gas phase methods, the detection of oxidized forms of NO is not possible. But after measuring NO by this method, the samples can be ground and further analyzed by using mass spectrometer, indirect chemiluminescence, Griess reagent or NO electrodes.

### Measurement of NO in the liquid phase

A common method of measuring NO in liquid phase is based on using the NO electrodes. These include platinum/teflon or platinum/iridium (Pt/Ir) coated working electrode and Ag/AgCl reference electrode. NO is detected via its oxidation at +0.8 to +0.9 V compared to the reference electrode (Shibuki, 1990). NO electrodes have been used in plant NO research, e.g., to measure NO production in tobacco cells in response to cryptogein (Besson-Bard et al., 2008), to detect NO in fruits (Leshem, 1996), to measure NADH-dependent NO scavenging activity in plant extracts and in purified fractions containing class 1 hemoglobin (Igamberdiev et al., 2004, 2006). The disadvantage of this method is that it measures NO in the liquid phase and if plant tissues emit NO in the gaseous phase, it is not quite reliable to detect it using these electrodes. The best combination is the use of the chemiluminescence method to detect NO in the gas phase and then NO electrodes to check NO concentration in the extracts of plant tissues.

As we have mentioned above, NO is highly reactive and only its small portion is emitted from the biological samples while the rest is oxidized (Vanin et al., 2004), therefore it is not possible to measure oxidized forms of NO such as nitrate, nitrite by gas phase chemiluminescence. But this limitation can be overcome by using indirect chemiluminescence in which nitrate and nitrite produced from oxidation of NO are reduced back to NO by injecting sample extracts into boiling acidic vanadium chloride (Gupta and Kaiser, 2010).

If there is no proper equipment for doing indirect chemiluminescence, then the alternative method to measure oxidized forms of NO is the Griess reagent assay, which is relatively cheap. In this method NO is oxidized to nitrite which reacts with sulphanilic acid and α-naphthylamine under acidic conditions to produce the azodye which can be detected at 520 nm. This method is not commonly used by plant scientists but there are few reports (Shirinova et al., 1993; Planchet et al., 2005). Sensitivity is very low for this method (0.5 μM). Vitecek et al. (2008) showed that by using two traps (one for gas phase and one for liquid phase) it is possible to measure NO both is gas and liquid phases. The main problem of this method is the interference of internal nitrite which concentrations are much higher than NO.

Electron-spin resonance (ESR) is a well-accepted method to detect NO in a liquid phase. This method is based on detection of unpaired electrons that exhibit resonance in opposite orientations. This method is very specific for NO detection and its limit is in the picomolar range (Weaver et al., 2005). But the ESR spectrometer is expensive and special expertise is needed for operation. It cannot measure the emitted NO but it rather detects the trapped NO, and the online measurement of NO is not possible by this method.

Another biochemical assay to measure NO is the oxyhemoglobin assay. It is based on the reaction of oxyhemoglobin (HbO_2_) with NO resulting in the production of methemoglobin (MetHb) and nitrate (NO^−^_3_). Methemoglobin is detected at 401 nm. This method has considerable sensitivity which is in the nanomolar range (Murphy and Noack, 1994). This assay has been used by plant scientists (Cvetkovska and Vanlerberghe, 2012). Although it has a good sensitivity, the serious problem is that the reactive oxygen species oxidize HbO_2_ and give false positive results. The changes in pH can also affect the reaction. Since both ROS production and pH change are a part of stress response, caution should be taken while interpreting the results used via this method. NO is a free radical molecule that escapes from the site of production to target and also diffuses to the atmosphere. By the time hemoglobin assays is done there is a huge possibility that NO escapes from the sample. The techniques like oxyhemoglobin assay should be coupled with another measurement method like chemiluminescence, which can measure NO in the gas phase.

A widely used and most controversial method for NO detection is diaminofluorescein (DAF) fluorescent dyes (Foissner et al., 2000; Lamotte et al., 2004; Corpas et al., 2006; Prats et al., 2008; Cvetkovska and Vanlerberghe, 2012 and many other references). The principle of this method is based on 4,5-diaminofluorescein diacetate (DAF-2DA) diffusion into cells where the acetate groups are removed by intracellular esterases and generate 4,5-diaminofluorescein; DAF-2 can also react with N_2_O_3_, an oxidation product of NO, to generate the highly fluorescent DAF-2T (triazolofluorescein).

## The use of fluorescent dyes: advantages and cautions

The advantage of DAF dyes that it is very easy to apply them and observe the NO fluorescence using the fluorescent or confocal microscope, which are easily available commercially and cost-effective. If NO is produced in specific sites such as guard cells, meristems or nodules or pathogen-infected cells these dyes can easily react with NO and give good indication about NO production. DAF dyes are relatively sensitive to NO having the detection limit in the nanomolar range and moreover no additional fluorescence is observed with NO^−^_2_, NO^−^_3_, H_2_O_2_, and ONOO^−^ (Kojima et al., 1998).

However, the application of fluorescent dyes has been challenged by various studies. For instance, DAF2 reacts with dehydroascorbic acid (DHA) and ascorbic acid (AA) and forms fluorescent products within the similar range of fluorescence as DAF-2T (Zhang et al., 2002). Jourd'heuil (2002) was the first who suggested that DAF fluorescence is sensitive to NO only in the presence of superoxide (O^−^_2_) or peroxynitrite (ONOO^−^). This was confirmed in the independent study by Roychowdhury et al. (2002). The value of pH can also affect the fluorescence from DAF (Vitecek et al., 2008). Planchet and Kaiser (2006) compared DAF fluorescence with the chemiluminescence method and found that tobacco cell suspension that produces DAF fluorescence does not necessarily produce NO signal in chemiluminescence. Rümer et al. (2012) have shown that the cyptogein induced tobacco cells produce multiple products that generate DAF-fluorescence *in vitro* which is not attributed to DAF-2T, some of them are attributed to the reaction of apoplastic peroxidase, DAF and H_2_O_2_ in which DAF was a substrate for peroxidase. Horseradish-peroxidase plus H_2_O_2_ also generated DAF-fluorescence *in vitro*.

Carboxy-PTIO (cPTIO) is a widely used NO scavenging compound to check whether NO is responsible for the observed fluorescence. The mechanism of cPTIO effect is based on the oxidation of NO to NO_2_, and thereby it scavenges NO. But when the excess of NO is produced, NO reacts with NO_2_ and forms N_2_O_3_ which leads to the increased fluorescence. Therefore, the cPTIO-based NO assessment depends on NO concentrations. Moreover Rümer et al. (2012) found that the decrease in DAF fluorescence by applying cPTIO does not necessarily indicate the initial presence of NO since cPTIO can also decrease H_2_O_2_ production. During various stress conditions plants produce NO and ROS. Therefore, the use of DAF fluorescent dye can hamper the actual situation of NO status. On the other hand, for determing NO production in specific cells such as root tips or stomatal guard cells, fluorescence indicators are necessary to distinguish NO producing cells from non-producing cells. Another frequently used dye is DAF-FMDA having higher sensitivity than DAF-2DA (3 nM). But the disadvantage of DAF-FMDA is that its fluorescence depends on pH (Vitecek et al., 2008). Since plant cells exhibit different pH values at different stress conditions, the DAF-FM method should be used with caution. Another fluorescence dye is diaminorhodamine-4M (DAR-4M) (Lacza et al., 2005) but it is useful for assessment of total reactive nitrogen species (RNS) rather than for measurement of NO.

## Conclusion

Taken together all the information presented here can teach us various things:

Fluorescent dyes are very useful for detecting NO in specific cells but, as described above, they have various disadvantages. It should be recommended to do independent measurement of DAF-2T using high-pressure liquid chromatography (Rümer et al., 2012). Always one should detect autofluorescence before adding fluorescent dyes to the samples. Only few authors used two methods for NO detection which provides necessary comparison and gives more reliable results. For instance, Planchet and Kaiser (2006) used DAF fluorescence and chemiluminescence; Bright et al. (2009) used DAF fluorescence and EPR; Cantrel et al. (2011) used DAF fluorescence and chemiluminescence assay; Gupta et al. (2013) used chemiluminescence and quantum cascade laser; DAF fluorescence and hemoglobin assay were used by Cvetkovska and Vanlerberghe (2012).

On the other hand, many studies still lacking the practice of using dual approach methods which results in low reliability because of the lack of independent verification of uncertainties generated by the restrictions of one method applied. Therefore, we recommend always using two independent methods with appropriate controls in order to obtain valuable information about NO concentrations and distribution in plant growth, development and stress response. In particular, we recommend using at least one gas phase method (1) and one liquid phase method (2) (See below).

**Table:** 

**Method 1 (gas phase)**	**Method 2 (liquid phase)**
Chemiluminescence	NO electrodes
Laser-based photoacoustic detection	Indirect chemiluminescence
Quantum cascade laser	Electron spin resonance
Membrane inlet mass spectrometry	Oxyhemoglobin assay
	Griess reagent assay
	Fluorescent based dyes
